# CO_2_ and O_2_ removal during continuous veno-venous hemofiltration: a pilot study

**DOI:** 10.1186/s12882-019-1378-y

**Published:** 2019-06-17

**Authors:** Joop Jonckheer, Herbert Spapen, Aziz Debain, Joy Demol, Marc Diltoer, Olivier Costa, Katrien Lanckmans, Taku Oshima, Patrick Honoré, Manu Malbrain, Elisabeth De Waele

**Affiliations:** 1 Intensive Care, UZ Brussel, Laarbeeklaan 101, 1090 Jette, Belgium; 2 Geriatrics, UZ Brussel, Laarbeeklaan 101, 1090 Jette, Belgium; 3 Department of Nutrition, Laarbeeklaan 101, 1090 Jette, Belgium; 4 Department of Clinical Laboratory, UZ Brussel, Laarbeeklaan 101, 1090 Jette, Belgium; 5 Emergency and Critical Care Medicine, Chiba University Graduate School of Medicine, 1-8-1 Inohana Chuo-ku, Chiba City, 260-8677 Japan; 6 Intensive Care, CHU Brugmann, A. Van Gehuchtenplein 4, 1020, Brussel, Belgium

**Keywords:** Continuous renal replacement therapy, Continuous veno-venous hemofiltration, Carbon dioxide removal, Oxygen removal, Citrate

## Abstract

**Background:**

Carbon dioxide (CO_2_) accumulation is a challenging issue in critically ill patients. CO_2_ can be eliminated by renal replacement therapy but studies are scarce and clinical relevance is unknown. We prospectively studied CO_2_ and O_2_ behavior at different sample points of continuous veno-venous hemofiltration (CVVH) and build a model to calculate CO_2_ removal bedside.

**Methods:**

In 10 patients receiving standard CVVH under citrate anticoagulation, blood gas analysis was performed at different sample points within the CVVH circuit. Citrate was then replaced by NaCl 0.9% and sampling was repeated. Total CO_2_ (tCO_2_), CO_2_ flow (V̇CO_2_) and O_2_ flow (V̇O_2_) were compared between different sample points. The effect of citrate on transmembrane tCO_2_ was evaluated. Wilcoxon matched-pairs signed rank test was performed to evaluate significance of difference between 2 data sets. Friedman test was used when more data sets were compared.

**Results:**

V̇CO_2_ in the effluent (26.0 ml/min) correlated significantly with transmembrane V̇CO_2_ (24.2 ml/min). This represents 14% of the average expired V̇CO_2_ in ventilated patients. Only 1.3 ml/min CO_2_ was removed in the de-aeration chamber, suggesting that CO_2_ was almost entirely cleared across the membrane filter. tCO_2_ values in effluent, before, and after the filter were not statistically different. Transmembrane tCO_2_ under citrate or NaCl 0.9% predilution also did not differ significantly. No changes in V̇O_2_ were observed throughout the CVVH circuit. Based on recorded data, formulas were constructed that allow bedside evaluation of CVVH-attributable CO_2_ removal.

**Conclusion:**

A relevant amount of CO_2_ is removed by CVVH and can be quantified by one simple blood gas analysis within the circuit. Future studies should assess the clinical impact of this observation.

**Trial registration:**

The trial was registered at https://clinicaltrials.gov with trial registration number NCT03314363 on October 192,017.

**Electronic supplementary material:**

The online version of this article (10.1186/s12882-019-1378-y) contains supplementary material, which is available to authorized users.

## Background

Red blood cells and plasma harbour carbon dioxide (CO_2_) in the form of dissolved CO_2_, bicarbonate, and carbamino compounds which are in equilibrium with each other [1]. The sum of all components is expressed as total CO_2_ (tCO_2_).

CO_2_ accumulation causes hypercarbia which may be a challenge in intensive care unit (ICU) patients. It has propagated a more extensive use of extracorporeal techniques to enable ultra-protective ventilation in acute respiratory distress syndrome or to avoid intubation in patients with severe exacerbation of chronic obstructive lung disease [2]. Although renal replacement therapy (RRT) is advocated to generate small amounts of CO_2_ due to the red blood cell passing through the filter [3], the net effect is a removal of CO_2_ in an intermittent hemodialysis (IHD) model with acetate [3, 4] This CO_2_ extraction of 41 ml/min seemed to correlate with a deficit of 46 ml/min in expired CO_2_ [5]. Different methods have been explored to increase the removal of CO_2_ in effluent by increasing pH with THAM or NaOH but it seemed too complex and too dangerous to be used in humans. [6] Extraction reached up to 120 ml/min in an in vitro model of IHD [7]. “CO_2_ loss” induced by RRT may become clinically relevant as mean expired CO_2_ in ICU patients is 180 ml/min [8]. Continuous RRT (CRRT) is progressively supplanting intermittent dialysis in the ICU. CRRT is hemodynamically well-tolerated, may provide easier control of metabolic alterations and fluid overload, and is associated with less chronic kidney disease in the post-ICU phase [9–11]. The impact of CRRT on CO_2_ metabolism is remarkably poorly documented. In addition, trisodium citrate - the preferred anticoagulant for CRRT- acts as a weak acid [12]. This will alter the Henderson-Hasselbalch equation, disrupt the balance between the different CO_2_ forms, and thus potentially influence CO_2_ extraction during CRRT.

We designed a study to better understand CO_2_ and O_2_ extraction during CRRT. Based on obtained data, formulas were constructed to assess CRRT-related CO_2_ clearance at the bedside.

## Methods

A prospective study was performed in critically ill patients undergoing continuous veno-venous hemofiltration (CVVH). The study was approved by the Ethical Committee of the University Hospital Brussels (reference BUN 143201731636) and registered at https://clinicaltrials.gov (reference NCT03314363). Informed consent was obtained from the patient or a legal representative.

CVVH was performed with the Prismaflex®(Lund, Sweden) Baxter® device equipped with a Prismaflex® Baxter®, AN69 surface treated (ST) filter of 1.5 square meter (Meyzieu, France). Prismocitrate®18/0 (Sondalo, Italy) Baxter® was used as predilution and Prismocal® B22 (Sondalo, Italy) Baxter® or NaCl 0.9% as postdilution fluid. Dosing and postdilution fluid use were initiated and adapted according to an implemented standard CVVH protocol.

**Fig. 1:**
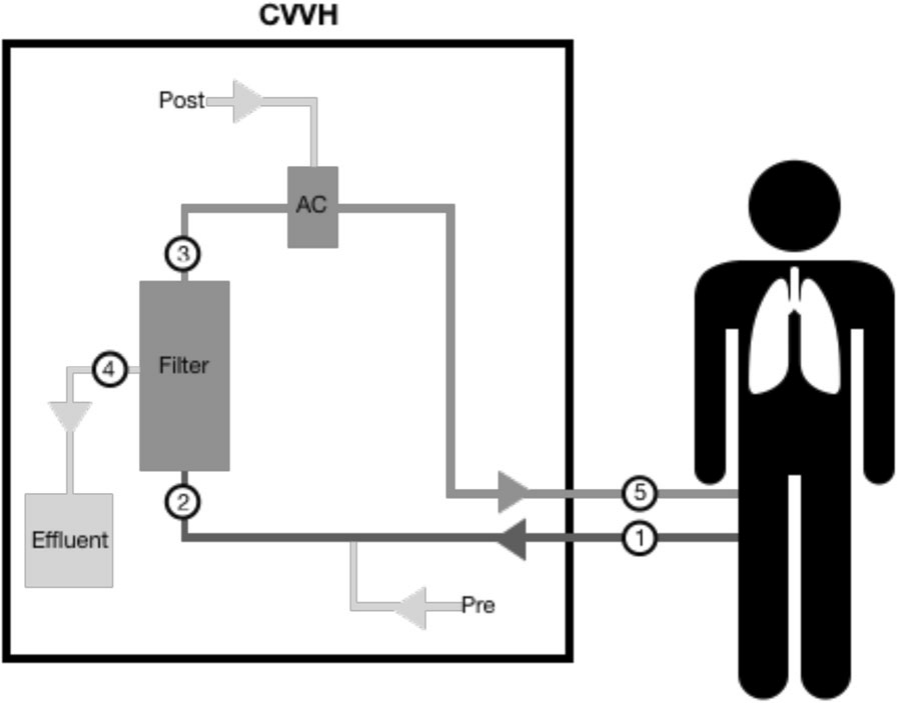
Schematic representation of CVVH set-up with location of sampling points and ports of fluid infusion. Pre = predilution fluid, Post = postdilution fluid, AC = de-aeration chamber

Inclusion and exclusion criteria are listed in Additional file 1. Blood samples were taken at 5 different sample points (SP) (Fig. 1). SP were located at the distal end of the arterial dialysis catheter lumen (SP1); between citrate predilution infusion port and filter (SP2); directly after the filter (SP3); at the effluent conduit (SP4); proximal to the venous dialysis catheter lumen (SP5). At every SP, pH, HCO_3_-, tCO_2_, pCO_2_, pO_2_, hemoglobin (Hb) and Hb saturation were measured using a blood gas analyzer [ABL90 Flex®, Radiometer(Bronshoj, Denmark)]. Subsequently, citrate predilution was stopped and replaced for at least 20 min by NaCl 0.9% at similar flow. Blood gas analysis was repeated according to the same protocol.

O_2_ content (tO_2_) was calculated as Hb x Hb saturation × 1.35 [1]. CO_2_ (V̇CO_2_) and O_2_ flow (V̇O_2_) at the specific SP were calculated by multiplying the set fluid flow (Q) on CVVH with respectively tCO_2_ and tO_2_. Results were adjusted from mmol to ml by using Boyle’s gas law: pV = nRT (p: pressure of the gas, V: volume of gas, n: amount of substance of gas, R: gas constant, T absolute temperature of the gas). The average air pressure recorded by the Belgian national weather institute was applied and ambient temperature was measured. “Transmembrane” (i.e before and after the filter) V̇CO_2_ was calculated by subtracting V̇CO_2_ at SP3 from V̇CO_2_ at SP2. “Transmembrane tCO_2_” was calculated in the same way.

tCO_2_ of bicarbonate fluid was 22 mmol/l, converted by gas law to ml/l depending on ambient conditions, and calculated in ml/min based on the flow set on CVVH. When bicarbonate was used, the “expected V̇CO_2_ at SP5” was calculated by adding the calculated V̇CO_2_ of the postdilution fluid to the V̇CO_2_ at SP3.

Relevant parameters such as CRRT settings were collected tobe used in a predictive equation.

### Statistical analysis

Data were analyzed using Prism Graphpad® version 7(La Jolla, USA). As data sets contained 18 values at the most, normality was not assessed. Data are expressed as mean ± standard deviation. Wilcoxon matched-pairs signed rank test was performed to evaluate significance of difference between 2 data sets. Friedman test was used when more data sets were compared. Differences in measured data, V̇CO_2_ and V̇O_2_ between SP were evaluated.

V̇CO_2_ in the effluent (SP4) was compared with “transmembrane V̇CO_2_”. “Expected V̇CO_2_ at SP5” was compared with V̇CO_2_ at SP5. A subgroup analysis was performed to compare the influence of citrate on CO_2_ extraction by the filter by comparing “transmembrane tCO_2_” with and without citrate.

## Results

Summary of patient characteristics are depicted in Table 1. CVVH settings of patients are provided in Additional file 2. Predilution citrate was not replaced by NaCl 0.9% in 2 patients because pre-existing hypercoagulability could compromise filter function.

**Table 1:** patient characteristics

Patients (n)	10
Age (years)	68.7 ± 11.3
Gender (male/female)	8/2
BMI (kg/m^2^)	29.8 ± 7.3
APACHE II	27.1 ± 9.0
Reason for admission:	
Medical	8
Surgical	2
Receiving controlled or assisted ventilation at day of study (n)	9
Mean CVVH settings in all series of blood gas analysis (ml/h)	
Bloodflow	9000 ± 0
Predilution	1750 ± 447
Postdilution	444 ± 170
Effluent flow	2380 ± 175

Data are presented as means ± standard deviation

### Comparison of V̇CO_2_

V̇CO_2 (SP1)_ was higher than V̇CO_2 (SP5)_ and V̇CO_2 (SP2)_ [111.3 ± 8.1 ml/min vs. respectively 87.4 ± 14.6 ml/min (*p* < 0.01) and 110.5 ± 9.6 ml/min (*p* = 0.03)]. V̇CO_2_ dropped significantly between SP2 and SP3 [from 110.5 ± 9.6 ml/min to 84.5 ± 6.5 ml/min (p < 0.01)]. V̇CO_2_ at SP4 (26.0 ± 5.8 ml/min) and transmembrane V̇CO_2_ at SP4 (24.2 ± 2.6 ml/min) were not statistically different (*p* = 0.39).

**Fig. 2:**
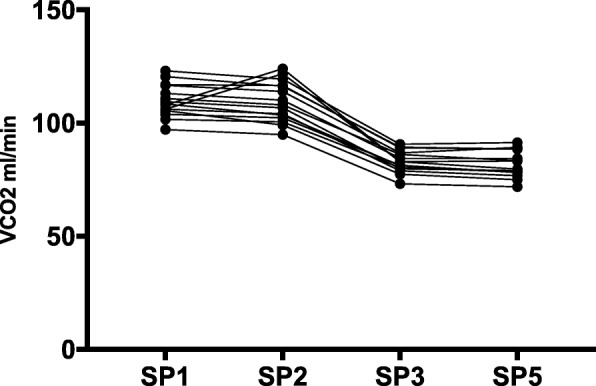
Evolution of CO_2_ flow in the extracorporeal blood circuit during NaCl 0.9% postdilution. Sample point 4 was not included as it is not situated in the extracorporeal blood circuit and is not suited to represent evolution of V̇CO_2_ in the blood. When difference between data was statistical significant different, this was marked with an asterisk

Results for NaCl 0.9% postdilution were plotted in Fig. 2. Patients receiving postdilution NaCl 0.9% exhibited higher V̇CO_2 (SP1)_ than V̇CO_2 (SP5)_ [109.8 ± 7.1 ml/min vs. 81.7 ± 5.8 ml/min (*p* < 0.01)] and a 1.3 ml/min difference between V̇CO_2 (SP3)_ and V̇CO_2 (SP5)_ [83.0 ± 4.9 ml/min vs. 81.7 ± 5.8 ml/min (*p* = 0.01)].

**Fig. 3:**
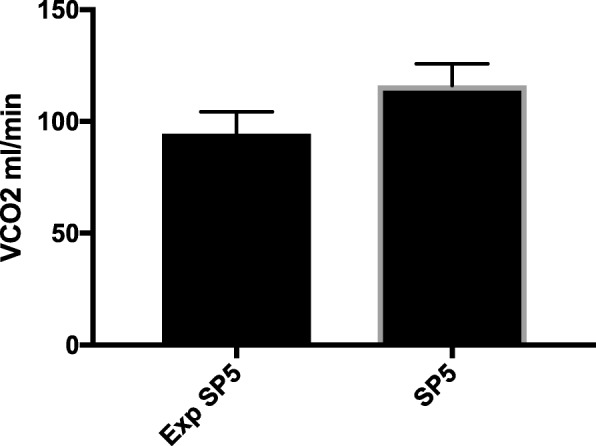
Expected CO_2_ flow in sample place 5 versus CO2 flow at sample place 5 during bicarbonate containing Prismocal B22® postdilution. When difference between data was statistical significant different, this was marked with an asterisk

No statistical analysis was performed when bicarbonate was used as postdilution fluid as it only consisted of 3 data sets. A 21.7 ml/min difference was noted between V̇CO_2_ at SP5 (116.1 ± 9.7 ml/min) and “expected V̇CO_2_ at SP5” (94.4 ± 9.9 ml/min) (Fig. 3).

### Comparison of tCO_2_

**Fig. 4:**
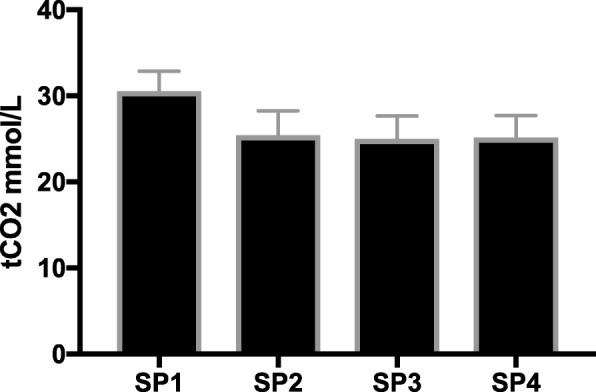
Evolution of tCO_2_ at different sample points in all series of blood gas analysis. Sample point 5 was not included as it is influenced by bicarbonate Prismocal B22 postdilution fluid. When difference between data was statistical significant different, this was marked with an asterisk

**Fig. 5:**
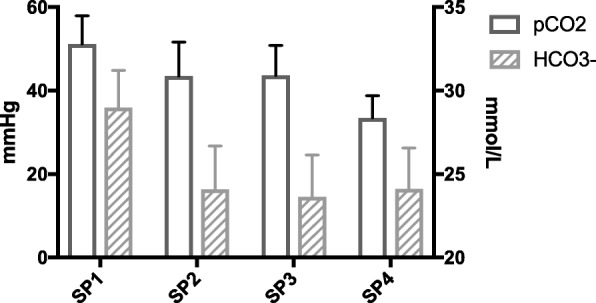
Distribution of pCO_2_ and HCO_3_- at different sample points in all series of blood gas analysis. Sample point 5 was not included as it is influenced by bicarbonate Prismocal B22 postdilution fluid

Results are given in Fig. 4. tCO_2_ at SP2 (25.5 ± 2.8 mmol/l), SP3 (25.0 ± 2.6 mmol/l) and SP4 (25.1 ± 2.6 mmol/l) were not statistically different (*p* = 0.51). tCO_2_ decreased significantly between SP1 and SP2 from 30.6 ± 2.3 mmol/l to 25.5 ± 2.8 mmol/l (p < 0.01). At all SP, tCO_2_ consisted of CO_2_ in gas form (pCO_2_) and HCO_3_- (Fig. 5).

### Effect of citrate vs. no-citrate predilution on transmembrane tCO_2_

**Fig. 6:**
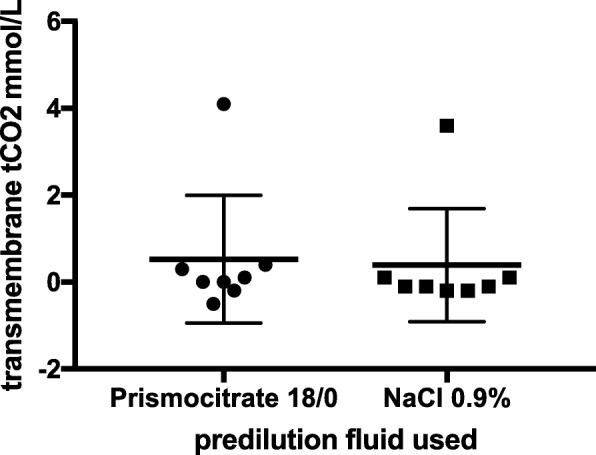
Effect of citrate on transmembrane tCO_2_

Patients in whom citrate could not be withdrawn were excluded from analysis. ΔtCO_2_ between SP2 and SP3 was not different in the citrate vs no-citrate group (*p* = 0.21) (Fig. 6).

### Comparison of V̇O_2_

**Fig. 7:**
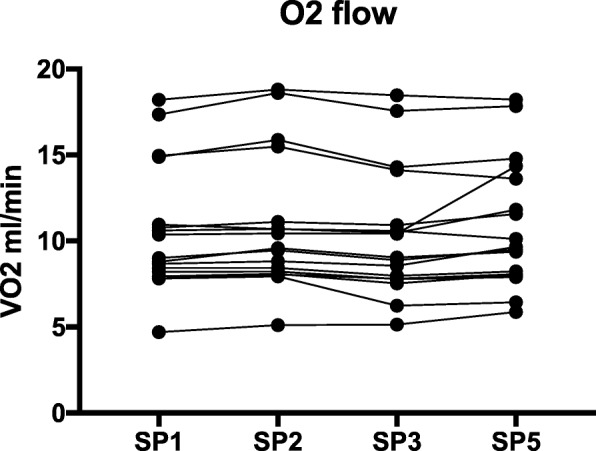
Evolution of O_2_ flow in the extracorporeal blood circuit in all series of blood gas analysis. Sample point 4 was not included as it is not situated in the extracorporeal blood circuit and is not suited to represent evolution of V̇O_2_ in the blood

V̇O_2_ at SP1, SP2, SP3 and SP5 was respectively 10.6 ± 3.7 ml/min,10.9 ± 3.9 ml/min, 10.3 ± 3.8 ml/min, and 10.9 ± 3.7 ml/min. V̇O_2_ at SP4 was 0 ml/min as effluent contains no Hb. V̇O_2 (SP1)_ and V̇O_2 (SP5)_ were not different (*p* = 0.33) (Fig. 7).

### Development of formulas

The above findings allow to propose following formulas:

$$ \dot{\mathbf{V}}\mathbf{C}{\mathbf{O}}_{\mathbf{2}\left(\mathbf{effluent}\right)}=\dot{\mathbf{V}}\mathbf{C}{\mathbf{O}}_{\mathbf{2}\left(\mathbf{SP}\mathbf{4}\right)}={\mathbf{Q}}_{\mathbf{SP4}}\ \mathbf{x}\ {\left[\mathbf{tC}{\mathbf{O}}_{\mathbf{2}}\right]}_{\mathbf{SP4}} $$

As tCO_2_ is similar at SP2, SP3 and SP4, the equation becomes:

$$ \dot{\mathbf{V}}\mathbf{C}{\mathbf{O}}_{\mathbf{2}\left(\mathbf{effluent}\right)}={\mathbf{Q}}_{\mathbf{SP4}}\ \mathbf{x}\ {\left[\mathbf{tC}{\mathbf{O}}_{\mathbf{2}}\right]}_{\mathbf{SP3}}={\mathbf{Q}}_{\mathbf{SP4}}\ \mathbf{x}\ {\left[\mathbf{tC}{\mathbf{O}}_{\mathbf{2}}\right]}_{\mathbf{SP2}}\ \left[{}^{\ast}\right] $$

By assuming that

$$ \dot{\mathrm{V}}{\mathrm{CO}}_{2\left(\mathrm{SP}1\right)}\approx \dot{\mathrm{V}}{\mathrm{CO}}_{2\left(\mathrm{SP}2\right)}<=>{\mathrm{Q}}_{\mathrm{SP}1}\ \mathrm{x}\ {\left[{\mathrm{tCO}}_2\right]}_{\mathrm{SP}1}\approx {\mathrm{Q}}_{\mathrm{SP}2}\ \mathrm{x}\ {\left[{\mathrm{tCO}}_2\right]}_{\mathrm{SP}2}<=>{\left[{\mathrm{tCO}}_2\right]}_{\mathrm{SP}2}\approx {\mathrm{Q}}_{\mathrm{SP}1}\ \mathrm{x}\ {\left[{\mathrm{tCO}}_2\right]}_{\mathrm{SP}1}/{\mathrm{Q}}_{\mathrm{SP}2} $$

When [tCO_2_]_SP2_ is substituted in the above formula [*], it becomes

$$ \dot{\mathbf{V}}\mathbf{C}{\mathbf{O}}_{\mathbf{2}\left(\mathbf{effluent}\right)}\approx {\mathbf{Q}}_{\mathbf{SP4}}\ \mathbf{x}\ {\mathbf{Q}}_{\mathbf{SP1}}\ \mathbf{x}\ {\left[\mathbf{tC}{\mathbf{O}}_{\mathbf{2}}\right]}_{\mathbf{SP1}}/{\mathbf{Q}}_{\mathbf{SP2}} $$

## Discussion

We present the first study that prospectively evaluated CO_2_ and O_2_ behavior in patients undergoing CVVH. The main finding was that a substantial amount of 26.0 ml/min CO_2_ was removed in the effluent. This represents approximately 14% of the average expired V̇CO_2_ measured in ICU patients [8] and thus could be clinically relevant. Furthermore, CO_2_ removal during CVVH was found to be 80% lower than previously observed in an in vitro hemodialysis model. This is explained by the almost threefold higher blood flow rate used in this model as compared to our CVVH setting [7]. V̇CO_2_ before and after predilution (Δ V̇CO_2_ between SP1 and SP2) was statistically different, probably because the set CVVH fluid flow at these SP did not correspond with real fluid flow [9]. Blood analysis also depended on “snapshot” sampling which might not exactly reflect average flow. However, this difference did not seem clinically relevant compared with average expired V̇CO_2_ (< 1%). CO_2_ flow was then divided between the effluent and the blood running to the de-aeration chamber (SP3 and SP4). The CO_2_ flow in the effluent correlated with V̇CO_2_ loss in the blood after passing the filter [$\dot{\mathrm{V}}{\mathrm{CO}}_{2\left(\mathrm{SP}2\right)}-\dot{\mathrm{V}}{\mathrm{CO}}_{2\left(\mathrm{SP}3\right)}=\dot{\mathrm{V}}{\mathrm{CO}}_{2\left(\mathrm{SP}4\right)}$].

In patients receiving postdilution NaCl 0.9%, 1.3 ml/min of CO_2_ was removed in the de-aeration chamber. This is a very small quantity compared to the average V̇CO_2_ in ICU patients [8]. Thus, CO_2_ removal was almost entirely determined by transmembrane filtering and measurable in the effluent. However, when postdilution bicarbonate was used, the expected V̇CO_2_ did not correspond with the calculated V̇CO_2_ in the blood before it re-entered the body. Several assumptions may explain this observation. First, measurements may be incorrect when CO_2_ fails to enter red blood cells after being infused in the postdilution fluid into the extracorporeal circuit. Second, tCO_2_ was calculated and not measured. Formulas for these calculations may not be applicable in a non-physiological state of bicarbonate-induced blood alkalinization. Studies measuring blood tCO_2_ are needed to elucidate this problem.

As suggested by in vitro hemodialysis, CO_2_ is removed in the effluent in gas form and as HCO_3_- [7]. The CO_2_ concentration or tCO_2_ is the driving force for this removal as it remains constant in effluent and in the blood passing through the filter [tCO_2(SP2)_ = tCO_2(SP3)_ = tCO_2(SP4)_]. By adding predilution fluid, tCO_2_ decreased between SP1 and SP2.

Citrate anticoagulation did not influence tCO_2_ extraction. Only the short term effect of citrate upon CO_2_ removal was evaluated as an influencer of acid-base homeostasis. Over a longer time period, citrate could possibly affect CO_2_ clearance because it preserves membrane porosity better than heparin. tCO_2_ in blood passing through the CVVH circuit decreased as it was diluted by bicarbonate-free solutions. CVVH had no impact on V̇O_2_ because values remained constant at the different SP.

Based on previous findings, different formulas were constructed to calculate CO_2_ removal by CVVH in a clinical setting with the use of only one blood gas analysis in the extracorporeal circuit at a preexisting sample point. As these are the first data that were acquired in a CVVH setting, formulas could not be compared to data from other articles [7]. Further studies need to confirm our findings.

Several limitations of our study must be emphasized. First, despite the high number of analyses per patient, the sample size remains small and future studies in more patients are needed to confirm our results. Second, assumptions were made based on “snapshot” blood gas analysis. Continuous monitoring would be more precise. Third, fluid flows as set on CVVH may not correlate with real flow [13]. In-circuit flow measurements may be a better option. Finally, it remains to be determined whether the removed CO_2_ influences expired CO_2_.

## Conclusion

A significant amount of CO_2_, both as gas and bicarbonate and measurable in the effluent, is removed during CVVH under citrate anticoagulation. Pre-filter tCO_2_ is the major determinant for CO_2_ removal. Citrate does not influence CO_2_ elimination. To a certain extent, bicarbonate fluids influence blood gases but data are too limited to permit relevant conclusions. Oxygen flow is not influenced by CVVH. CO_2_ removal by CVVH in bicarbonate-free conditions can be calculated by multiplying effluent or blood flow with CO2 content at a preexisting sample point. Their clinical relevance requires confirmation.

## Additional files

Additional file 1: Inclusion and exclusion criteria that were used during the study. (DOCX 15 kb)

Additional file 2: CVVH settings and postdilution fluid per patients. (DOCX 14 kb)

## Abbreviations

CO_2_: Carbon dioxide
CRRT: Continuous renal replacement therapy
CVVH: Continuous veno-venous hemofiltration
Hb: Hemoglobin
ICU: Intensive care unit
IHD: Intermittent hemodialysis
n: Amount of substance of gas
O_2_: Oxygen
p: Pressure of the gas
Q: Fluid flow
R: Gas constant
RRT: Renal replacement therapy
SP: Sample point
T: Absolute temperature of the gas
tCO_2_: Total CO_2_
tO_2_: Oxygen content
V: Volume of gas
V̇CO_2_: CO_2_ flow
V̇O_2_: O_2_ flow
