# Correction to: CO_2_ and O_2_ removal during continuous veno-venous hemofiltration: a pilot study

**DOI:** 10.1186/s12882-019-1480-1

**Published:** 2019-08-08

**Authors:** Joop Jonckheer, Herbert Spapen, Aziz Debain, Joy Demol, Marc Diltoer, Olivier Costa, Katrien Lanckmans, Taku Oshima, Patrick M. Honoré, Manu Malbrain, Elisabeth De Waele

**Affiliations:** 1 Intensive Care, UZ Brussel, Laarbeeklaan 101, 1090 Jette, Belgium; 2 Geriatrics, UZ Brussel, Laarbeeklaan 101, 1090 Jette, Belgium; 3 Department of Nutrition, Laarbeeklaan 101, 1090 Jette, Belgium; 4 Department of Clinical Laboratory, UZ Brussel, Laarbeeklaan 101, 1090 Jette, Belgium; 5 Emergency and Critical Care Medicine, Chiba University Graduate School of Medicine, 1-8-1 Inohana Chuo-ku, Chiba City, 260-8677 Japan; 6 Intensive Care, CHU Brugmann, A. Van Gehuchtenplein 4, 1020 Brussel, Belgium

**Correction to: BMC Nephrology (2019) 20:222**

**https://doi.org/10.1186/s12882-019-1378-y**

Following publication of the original article [1], the authors reported that one of the authors’ names was spelled incorrectly. In this Correction the incorrect and correct author name are shown. The original publication of this article has been corrected.

Originally the author name was published as:

- Patrick Honoré

The correct author name is:

- Patrick M. Honoré
