# Supplementary material for: CO2 and O2 removal during continuous veno-venous hemofiltration: a pilot study
Source: BMC Nephrol. 2019 Jun 17;20:222. doi: 10.1186/s12882-019-1378-y (PMC6580471; doi:10.1186/s12882-019-1378-y)
Supplement: Supplementary file 1 — Inclusion and exclusion criteria that were used during the study. (DOCX 15 kb) [file 12882_2019_1378_MOESM1_ESM.docx]

**Additional file 1**

Inclusion criteria

- Male and female ICU patients, aged > 18 years
- AKI requiring CVVH
- Stability (approximately 2h) during study :
  - unchanged medication
  - unchanged respiratory conditions
  - stable pH (7.30-7.50) and lactate (< 2.0 mmol/l)
  - no interventions (transport, nursing care, physiotherapy)
  - unchanged CVVH settings
- Maximally allowed respiratory settings: FiO2: 60% ;inspiratory plateau pressure 30 cmH20; tidal volume 8ml/kg
- CVVH set-up:
  - blood pump flow: 150 ml/min
  - predilution (citrate): 1500-2300 ml/h
  - dialysate dose: 25-40 ml/kg/h
  - ultrafiltration rate: 0-300 ml /h
  - substitution: NaCl 0.9% 300-800 ml/h or Prismocal® B22: 400-2000 ml/h

Exclusion criteria

- Pregnancy / lactation
- Severe hemodynamic or respiratory instability.
- CVVH modalities not conform with routine practice
