# Supplementary material for: CO2 and O2 removal during continuous veno-venous hemofiltration: a pilot study
Source: BMC Nephrol. 2019 Jun 17;20:222. doi: 10.1186/s12882-019-1378-y (PMC6580471; doi:10.1186/s12882-019-1378-y)
Supplement: Supplementary file 2 — CVVH settings and postdilution fluid per patients. (DOCX 14 kb) [file 12882_2019_1378_MOESM2_ESM.docx]

| Patient | Blood flow  (ml/h) | Predilution flow  (ml/h) | Effluent flow  (ml/h) | Postdilution flow (ml/h) | Postdilution fluid |
| --- | --- | --- | --- | --- | --- |
| 1 | 9000 | 1700 | 2170 | 400 | NaCl 0.9% |
| 2 | 9000 | 1100 | 2400 | 400 | NaCl 0.9% |
| 3 | 9000 | 1750 | 2200 | 400 | NaCl 0.9% |
| 4 | 9000 | 1700 | 2250 | 350 | Prismocal® B22 |
| 5 | 9000 | 2000 | 2450 | 400 | Prismocal® B22 |
| 6 | 9000 | 1900 | 2500 | 400 | NaCl 0.9% |
| 7 | 9000 | 1850 | 2350 | 400 | NaCl 0.9% |
| 8 | 9000 | 1800 | 2400 | 600 | NaCl 0.9% |
| 9 | 9000 | 1900 | 2500 | 400 | NaCl 0.9% |
| 10 | 9000 | 1850 | 2700 | 800 | NaCl 0.9% |

**Additional file 2: CVVH Settings in different patients**
